# All that Doesn’t Enhance Isn’t a Thrombus: Pitfalls Using Cardiac MRI TI 600 Sequence to Distinguish Between Cardiac Thrombus Versus Myxoma

**DOI:** 10.14797/mdcvj.1322

**Published:** 2024-04-10

**Authors:** Aakash D. Rana, Srikanth Vallurupalli, Mark Mitchell, David Duncan, Jack Xu

**Affiliations:** 1 Central Arkansas Veteran Affairs Health System, Little Rock, Arkansas, US; 2 University of Arkansas for Medical Sciences, Little Rock, Arkansas, US; 3 Novant Health Forsyth Medical Center, Winston-Salem, North Carolina, US

**Keywords:** cardiac thrombus, myxoma, TI 600, CMR

## Abstract

A 51-year-old male with a complicated medical history presented with shortness of breath. Preoperative workup confirmed the presence of a large atrial mass. However, delayed gadolinium enhancement CMR with long inversion time (TI 600) showed lack of enhancement, which was suggestive of a thrombus. During cardiac magnetic resonance imaging, delayed gadolinium enhancement sequences with long inversion time (TI 600) are commonly used to distinguish between an avascular thrombus versus a vascular tumor.

## Description

**Figure 1:**
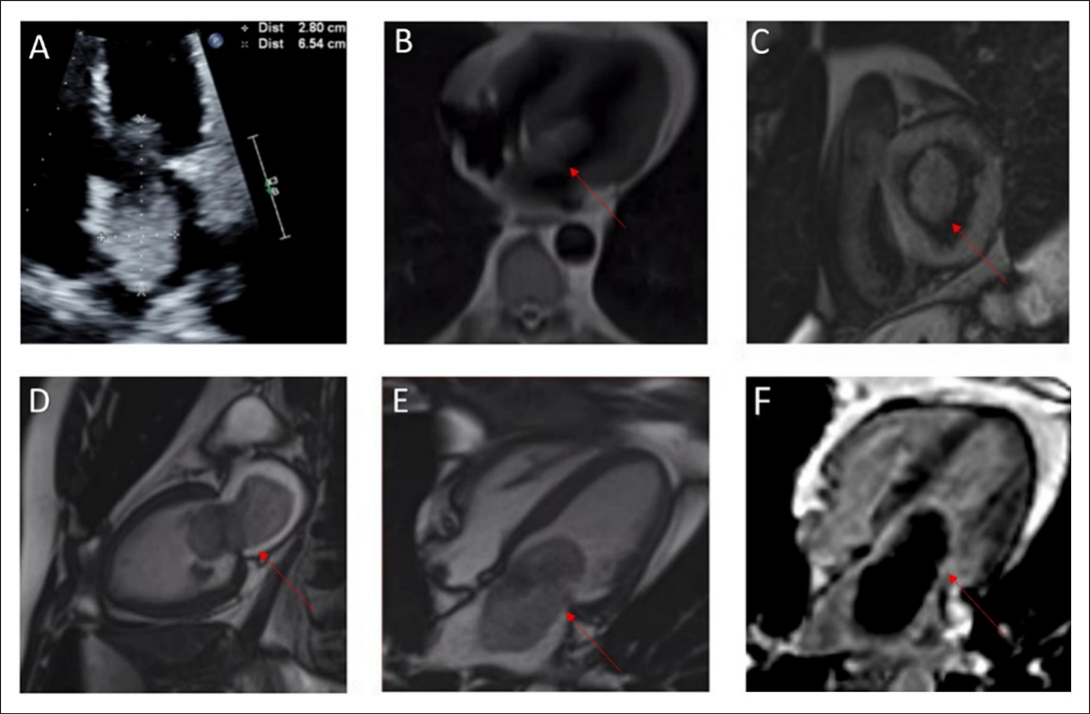
**(A)** Transthoracic echocardiogram of normal LV systolic function with an ejection fraction of 55% to 60% and large mobile echo density in the left atrium attached to the atrial septum; **(B-E)** cardiac magnetic resonance (CMR) imaging obtained for further characterization of the cardiac mass confirmed the presence of a large atrial mass attached to the fossa ovalis; **(F)** delayed gadolinium enhancement CMR with long inversion time suggested a thrombus.

A 51-year-old male with past medical history of hypertension, hyperlipidemia, coronary artery disease, and chronic obstructive pulmonary disease presented to the emergency department (ED) for shortness of breath. A computed tomography pulmonary angiogram (CTPE) was obtained due to concern for pulmonary embolism. The CTPE showed a filling defect in the left atrium straddling the mitral valve. Transthoracic echocardiogram showed normal left ventricular systolic function with an ejection fraction of 55% to 60% and large mobile echo density in the left atrium attached to the atrial septum (Figure 1 A). Cardiac magnetic resonance (CMR) imaging was obtained for further characterization of the cardiac mass (Figure 1 B-E), and it confirmed the presence of a large atrial mass attached to the fossa ovalis measuring 6.9 cm × 3.6 cm. Delayed gadolinium enhancement CMR with long inversion time (TI 600) showed lack of enhancement, which was suggestive of a thrombus (Figure 1 F).

During surgery, a 7 cm × 4 cm cardiac mass extending from the left atrium to the left ventricle was resected. The mass was associated with a significant thrombus burden on visual examination. Pathology report was consistent with myxoma with hemosiderin deposits.

This case highlights the importance of incorporating all clinical and imaging data in the diagnosis of a cardiac mass. The diagnostic value of the TI 600 sequence relies on the lack of vascularity of a thrombus compared with a vascular tumor. However, there are two common pitfalls with this paradigm. First, the specificity of the TI 600 sequence for diagnosis of thrombus is not 100% because some tumors (such as cardiac cysts) are relatively avascular. In one of the largest series published, up to 2% of tumors display lack of enhancement with TI 600 sequence.^1^ Furthermore, large tumors may outgrow their vascular supply and become necrotic. Second, organized thrombus can develop neovascularization and may enhance on TI 600 sequences.^2^ TI 600 sequences should be incorporated into the overall assessment of cardiac masses and not be solely used to differentiate between tumor and thrombus.
